# Intra-articular injection of kartogenin-conjugated polyurethane nanoparticles attenuates the progression of osteoarthritis

**DOI:** 10.1080/10717544.2018.1461279

**Published:** 2018-04-18

**Authors:** Wenshuai Fan, Jinghuan Li, Liu Yuan, Jifei Chen, Zhe Wang, Yiming Wang, Changan Guo, Xiumei Mo, Zuoqin Yan

**Affiliations:** a Department of Orthopedics, Zhongshan Hospital, Fudan University, Shanghai, China;; b Department of Hepatic Oncology, Liver Cancer Institute, Zhongshan Hospital, Fudan University, Shanghai, China;; c Biomaterials and Tissue Engineering Lab, College of Chemistry, Chemical Engineering and Biotechnology, Donghua University, Shanghai, China;; d State Key Laboratory for Modification of Chemical Fibers and Polymer Materials, College of Materials Science and Engineering, Donghua University, Shanghai, China

**Keywords:** Osteoarthritis, intra-articular injection, kartogenin, polyurethane nanoparticles

## Abstract

Osteoarthritis (OA) is the most common form of joint disease and a leading cause of physical disability, there is an urgent need to attenuate the progression of OA. Intra-articular (IA) injection is an effective treatment for joints diseases, however, the therapeutic effects mostly depend on the efficacy of drug duration in joints. Drug delivery system can provide drug-controlled release and reduce the number of IA injection. In this study, amphiphilic polyurethanes with pendant amino group were synthesized and amide bonds were formed between the amine group of polyurethane and the carboxyl group of kartogenin (KGN), a small molecular reported to show both regenerative and protective effects on cartilage. Our results showed that KGN-conjugated polyurethane nanoparticles (PN-KGN) were spherical and regular in shape with an average size of 25 nm and could sustained and controlled release of KGN *in vitro*. PN-KGN showed no cytotoxicity and pro-inflammatory effects on chondrocytes. The therapeutic effects in OA model showed that IA injection of KGN could attenuate the progress of OA, however, the cartilage degeneration became obviously at 12 weeks with matrix loss and vertical fissures. By contrast, IA injection of PN-KGN showed less cartilage degeneration with significant lower OARSI scores even at 12 weeks, indicating PN-KGN could further arrest the development of OA. Immunohistochemistry also validated that IA injection of PN-KGN retained the normal compositions of cartilage matrix, with much stronger Col II staining and less Col I staining. In conclusion, IA injection of PN-KGN is a better potential strategy to treat OA, with long-time cartilage protection and less IA injections.

## Introduction

Osteoarthritis (OA) is the most common form of chronic joint disease affecting 250 million people worldwide and anticipated to be the fourth leading cause of physical disability by the year 2020 with increasing aging population (Woolf & Pfleger, 2003; Loeser et al., 2016). It is characterized by progressive degeneration of cartilage and subchondral bone changes. As cartilage is avascular, alymphatic and aneural tissues, and chondrocytes are highly differentiated cells with poor proliferation and migration potential, cartilage has a limited capacity for self-repair and regeneration (Li et al., 2017). It is therefore a challenge to attenuate the progression of OA and promote the regeneration of cartilage.

Recently, a small molecular, kartogenin (KGN), was reported to show both regenerative effects and protective effects on cartilage by mediating the CBF-β/Runx1 signaling pathway (Johnson et al., 2012). Xu et al. (2015) reported that intra-articular (IA) injection of KGN promoted cartilage repair and increased hyaline-like cartilage formation in New Zealand white rabbits with full-thickness cartilage defects in the femur. Another study demonstrated that IA injection of KGN appeared to reduce cartilage degradation and prevented subchondral bone changes in a rat OA model (Mohan et al., 2016). However, the frequency of IA injection was once a week in these above-mentioned studies, and frequent IA injections may increase the possible risk of joint infection, which will aggravate the progression of OA (Chambers et al., 2017). Reducing the frequency of joint injection and prolonging the efficacy of KGN can be beneficial to clinical application.

Drug delivery system can provide drug-controlled release and prolong drug activity in order to reduce the number of medication applications (Pacardo et al., 2015). Amphiphilic polymers which can self-assemble into nanoparticles in solvent have been reported and explored for their use in drug delivery to enhance the aqueous solubility of water-insoluble therapeutic agents (Qiu et al., 2015). Polyurethanes are important amphiphilic polymers and carry urethane or carbamate bonds (–NH–COO–) in their main chains, having been widely used in regenerative medicine and drug-controlled release due to good biocompatibilities and tailorable molecule structures (Cherng et al., 2013; Ou et al., 2014).

In the present study, in order to react with drug molecules with carboxyl group(s), such as KGN, amphiphilic polyurethanes with pendant amino group were synthesized. The characterization of KGN conjugated polyurethane nanoparticles (PN-KGN) for sustained release *in vitro* and treatment for cartilage regeneration in OA joint *in vivo* were evaluated. We hypothesize that PN-KGN can further attenuate the progression of OA and promote the regeneration of cartilage in the case of reduced IA injections.

## Materials and methods

### Synthesis of polyurethane nanoparticles (PN) and KGN-conjugated PN (PN-KGN)

**Figure 1.**
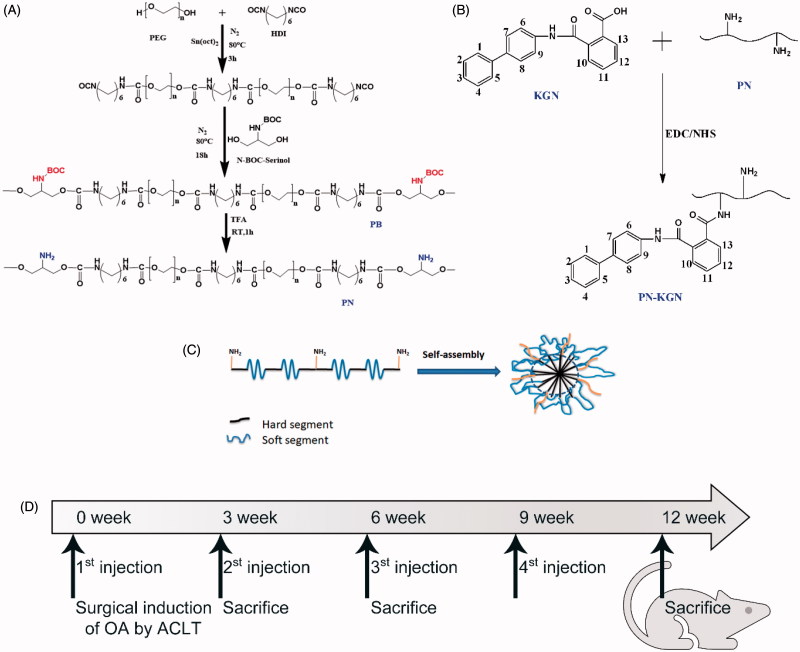
Illustration of the procedures to synthesize polyurethane (PN) and kartogenin (KGN) conjugated PN (PN-KGN) nanoparticles and structures of PN and PN-KGN (A–C). General scheme of *in vivo* experimental procedures in the surgically induced rat OA model. The rats were randomized into four groups: Control group, IA injections of saline; PN group, IA injections of PN; KGN group, IA injections of KGN; and PN-KGN group, IA injections of PN-KGN. IA injections were performed every three weeks at 0, 3, 6, and 9 weeks. Rats were sacrificed for analysis at 3, 6, and 12 weeks (D).

The polymers were synthesized from poly-(ethylene glycol) (PEG), hexamethylene diisocyanate (HDI) and N-BOC-Serinol with molar ratio of 1:2:1, followed by the further deprotection process of BOC-protected amino groups, which was performed as previously described method (Fang et al., 2014). Briefly, under nitrogen protection, 2 g PEG (1000 Da; 2 mmol) and 0.67 g HDI (4 mmol) were mixed in 20 mL DMSO in a three-necked flask, followed by addition of 0.05 wt% Sn(Oct)z. The reaction was carried out at 80 °C for 3 h and then cooled at room temperature. 0.382 g N-BOC-Serinol (2 mmol) in DMSO solution were dropwise added to the prepolymer solution. The final concentration of polymer solution was 5% (w/v) and the reaction continued at 80 °C for 18 h with stirring. After that, the mixture was precipitated in diethyl ether, and the resulting polymers were purified by dissolving in chloroform and further precipitating with diethyl ether for three times, dried in a vacuum oven at 45 °C for 2 days to obtain PB. To generate amphiphilic polyurethane with pendant amino groups (PN), synthesized PB (3 g) were dissolved to a 50% (w/v) concentration in 6 mL anhydrous chloroform/trifluoroacetic acid (TFA) (50/50) in a round bottom flask and stirred at room temperature for 1 h to remove the BOC-protected groups. After reaction, the excess anhydrous chloroform and TFA were moved through rotary evaporation. The polymers were further purified by dissolving in chloroform and precipitating with diethyl ether for three times. After that, the precipitates were dissolved and neutralized in 2% (w/v) NaHCO_3_ aqueous solution (pH = 8.3) to make sure TFA remove clearly. Then purified by dialysis (Mw = 3500 Da) against deionized water, and lyophilized (Figure 1(A)).

PN was grafted KGN through N-(3-dimethylaminopropyl)-N′-ethylcarbodiimide hydrochloride (EDC)/N-hydroxysuccinimide (NHS) condensation reaction to synthesize PN-KGN. First, NHS and EDC were added to KGN in DMSO to activate carboxylic acid groups (molar ratio of KGN: EDC: NHS is 3:6:2). After 30 min, the PN/DMSO solution was dropwise added to KGN solution, followed by stirring at room temperature for 24 h in dark. Then the products were dialyzed (Mw = 3500 Da) and lyophilized for 24 h (Figure 1(B)).

### Characterization of nanoparticles

Both Fourier transform infrared spectroscopy (FTIR) and proton nuclear magnetic resonance spectroscopy (^1^H NMR) were used to characterize the surface chemistry of the synthesized PN-KGN. The lyophilized powders of PN-KGN were applied on the FTIR sample folder and recorded on Nicolet 6700 FTIR spectrometer (Thermo Scientific, Waltham, MA). ^1^H NMR spectra were obtained using an Avance 400 NMR spectrometer (Bruker, Fällanden, Switzerland). The mean diameter and PDI of nanospheres were determined by dynamic laser light scattering (DLS, B1-200SM, Brookhaven, NY). The morphology of nanospheres was observed by transmission electron microscope (TEM, JEM-2100F, Jeol, Japan) operating at 200 kV.

### In vitro release study

The PN-KGN nanospheres (10 mg) were placed in 3 mL saline at 37 °C in a shaking incubator (100 rpm). The saline was collected after centrifugation (14,000 rpm, 15 min) and replaced with saline at each sampling time point. The amounts of released KGN in the collected saline were measured by reverse-phase high-performance liquid chromatography (HPLC; 600E-2487, Waters, Milford, MA) spectrum using a C-18 column (150 × 4.6 mm, 5 µm). The analysis was carried out with a flow rate of 1.0 mL/min and recorded at 274 nm for 30 min. The calibration curve for KGN was established in the range 2–200 mg/L.

### Isolation and culture of chondrocytes

Primary chondrocytes were obtained from Sprague Dawley (SD) rats. The articular cartilage derived from the terminal of femur and tibia was digested with 0.2% collagenase II (Sigma-Aldrich, St. Louis, MO) at 37 °C for 4 h. Digested chondrocytes were filtered through a 70 µm nylon filter (Becton Dickinson, Franklin Lakes, NJ) and plated into T25 flasks in growth medium which consisted of DMEM with 10% FBS (Gibco, Invitrogen, Carlsbad, CA) as passage 0. The cells were incubated at 37 °C in a humidified atmosphere with 5% CO_2_ and medium was replaced twice per week. The cells at passage 3 were utilized for subsequent experiments.

### Cytotoxicity test

The cytotoxicity of PN-KGN nanospheres on cell viability was evaluated by the Cell Counting Kit-8 (CCK-8) assay (Beyotime, Jiangsu, China). Briefly, passage 3 chondrocytes were plated in 96-well plates at a density of 5000 cells/well and then treated with different amounts of particles that can respectively maximally release KGN to 0, 10, and 100 nM according to the release curve. After incubation for 4 or 7 days with the nanospheres, the absorbance was measured using a spectrophotometric reader (SpectraMax 384, Sunnyvale, CA) at 450 nm. Cell numbers were compared to non-treatment control (*n* = 3).

### Pro-inflammatory activity

The pro-inflammatory activity of PN-KGN nanospheres was evaluated by measuring cytokines secreted from chondrocytes. Briefly, passage 3 chondrocytes were plated in six-well plates at a density of 1 × 10^5^ cells/well and then treated with lipopolysaccharide (LPS; 1 µg/mL), PN, and PN-KGN that can release KGN to 100 nM. The mediums were collected at each time point and concentrations of secreted interleukin-6 (IL-6) in the culture medium were assessed using the enzyme-linked immunosorbent assay (ELISA) according to the manufacturer’s instructions (R&D, Minneapolis, MN).

### Animals and study design

Ten-week-old male SD rats used in the animal experiments were provided by the Animal Research Committee of Zhongshan Hospital, Fudan University. All procedures were carried out according to the guide for the care and use of laboratory animals. OA was surgically induced by anterior cruciate ligament transection (ACLT) and destabilization of the medial meniscus according to previous reports (Hayami et al., 2006). The rats were randomized into four groups: Control group, IA injections of 100 µL saline; PN group, IA injections of 100 µL saline with PN (1.18 mg/mL); KGN group, IA injections of 100 µL saline with 100 µM KGN; and PN-KGN group, IA injections of 100 µL saline with PN-KGN (1.18 mg/mL) that can release 100 µM KGN (*n* = 6 per group). IA injections were performed every 3 weeks at 0, 3, 6, and 9 weeks. Rats were sacrificed for analysis at 3, 6, and 12 weeks after OA induction (Figure 1(D)).

### Macroscopic examination

After euthanasia, the surrounding soft tissues were removed and knee joints were carefully dissected without damaging the cartilage. The cartilage surface was then fully exposed and examined macroscopically. Macroscopic pictures were recorded by a digital camera (M205FA, Leica, Wetzlar, Germany).

### Histology

The dissected samples were fixed in 4% paraformaldehyde for 1 day and then decalcified in 10% EDTA for 6 weeks. After serial dehydration, the joints were embedded in paraffin and sagittally sectioned at 5 μm thickness. The sections were stained with safranin-O/fast green. The Osteoarthritis Research Society International (OARSI) cartilage histopathology assessment system was used to evaluate the degenerative status and OARSI scores were calculated using the formula: Score = Grade × Stage (Pritzker et al., 2006). Immunohistochemistry was performed to evaluate the metabolism of articular chondrocytes. Anti-type II collagen and anti-type I collagen mouse monoclonal antibodies (Novus, Littleton, CO) were used as primary antibodies (1:100). Detailed procedures of immunohistochemistry are described in our previous study (Fan et al., 2016).

### Statistical analysis

Statistical analyses were performed by GraphPad Prism (GraphPad Software Inc, La Jolla, CA). All data were expressed as mean ± standard deviation (SD). One-way analysis of variance (ANOVA) was used to assess group differences. *p* < 0.05 was considered statistically significant from at least three independent experiments.

## Results

### Synthesis of PN-KGN

**Figure 2.**
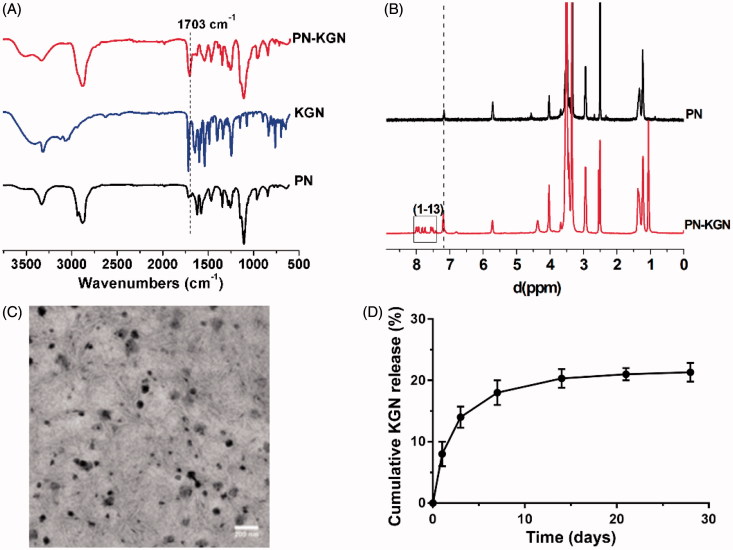
Typical FTIR (A) and ^1^H NMR spectra (B) showing the successful crosslink formation between PN and KGN, and the enhanced peaks at 1703 cm^−1^ (A) and 7.2 ppm (B) indicating the formation of amide bonds in PN-KGN. Transmission electron micrographs of PN-KGN, bar = 200 nm (C). *In vitro* release of KGN from PN-KGN at 37 °C (D).

Successful conjugation of PN-KGN was confirmed by FTIR and ^1^H NMR. The FTIR spectrum of PN-KGN showed enhanced peaks at 1703 cm^−1^ compared to that of PN, indicating the formation of amide bonds. At the same time, there were no absorption bands of ammonium salt (N–H) at 3200–2500 and 2150 cm^−1^, respectively, indicating that the conjugation between PN and KGN was not the formation of ammonium salt (Figure 2(A)). The ^1 ^H NMR spectra of PN-KGN showed the major peaks of the benzene rings in KGN at 7.3–7.9 ppm along with enhanced peaks of amide bonds at 7.2 ppm (Figure 2(B)). These results confirmed the conjugation of PN and KGN via the formation of amide bonds.

### Characterization of PN-KGN nanoparticles

TEM and DLS were used to characterize the morphology and determine the size of PN-KGN. The particles were spherical and regular in shape with an average size of 25 nm (Figure 2(C)). Figure 2(D) showed the sustained and controlled release of KGN from PN-KGN *in vitro*.

### Cytotoxicity and pro-inflammatory activity test

**Figure 3.**
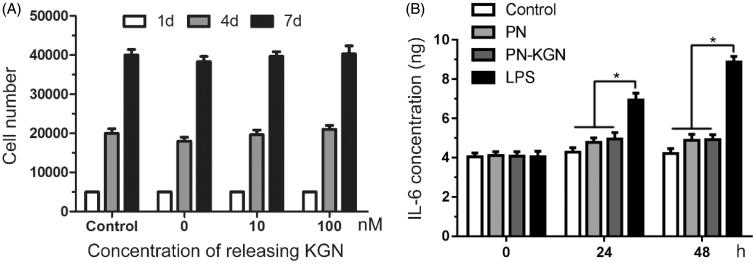
Cytotoxicity tests in chondrocyte with various doses of PN-KGN that can maximally release KGN to 0, 10, and 100 nM. The cell numbers were detected by CCK-8 at days 1, 4, and 7 (A). IL-6 secretions from chondrocytes were detected by ELISA after incubation with LPS (1 µg/ml), PN and PN-KGN that can release KGN to 100 nM (B). Data were shown as mean ± SD. *N* = 3, *p < 0.05.

The cytotoxicity of PN-KGN nanospheres was evaluated in chondrocytes. Figure 3(A) showed the amounts of chondrocytes after being exposed to the different amounts of PN-KGN that could release KGN from 0 to 100 nM. No significances were observed compared to the untreated chondrocytes at 4 and 7 days. The result indicated that both PN and PN-KGN nanoparticles had no cytotoxicity in chondrocytes.

The pro-inflammatory activity of PN-KGN nanospheres was also evaluated in chondrocytes. LPS treatment significantly increased the secretion of inflammatory cytokine IL-6 with time. However, addition of PN or PN-KGN did not increase the secretion of IL-6 compared to the untreated chondrocytes at 24 and 48 h. (Figure 3(B)). The result indicated that PN-KGN nanoparticles had no pro-inflammatory effects on chondrocytes *in vitro*.

### In vivo cartilage regeneration

**Figure 4.**
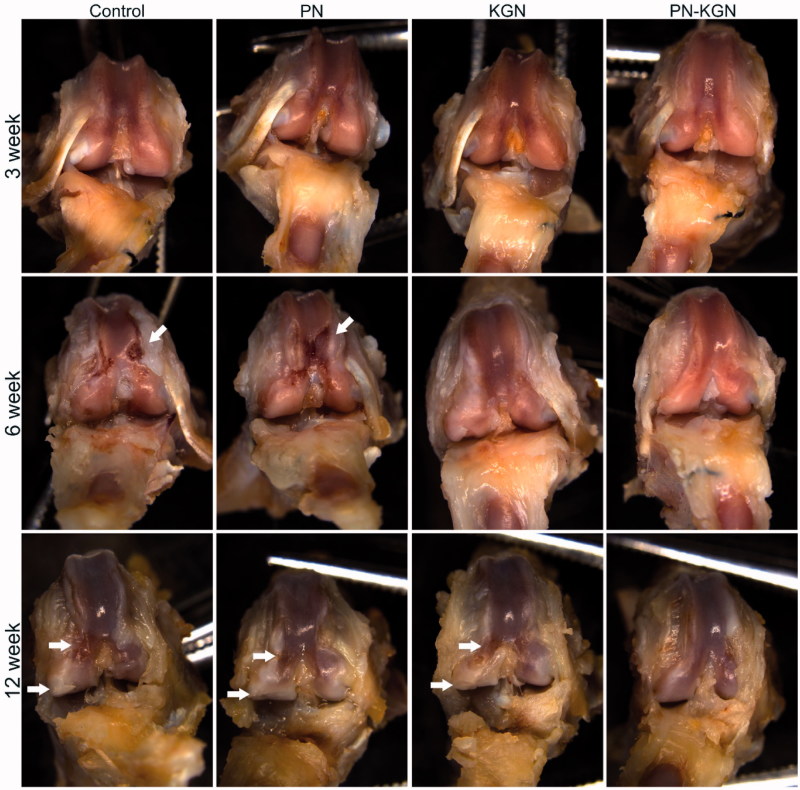
Macroscopic views of OA development at 3, 6, and 12 weeks after OA induction in four groups: Control group, IA injections of 100 µL saline; PN group, IA injections of 100 µL saline with PN; KGN group, IA injections of 100 µL saline with 100 µM KGN; and PN-KGN group, IA injections of 100 µL saline with PN-KGN that can release 100 µM KGN (*n* = 6 per group). Arrows indicate the locations of cartilage degeneration.

**Figure 5.**
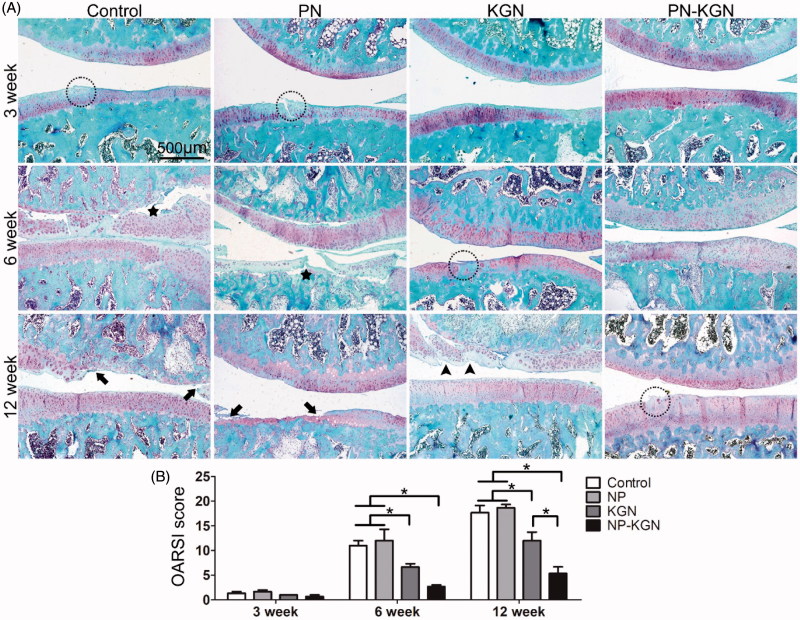
Safranin-O/fast green staining of OA progress at 3, 6, and 12 weeks after OA induction in Control group, PN group, KGN group and PN-KGN group. Stars: delamination of superficial layers, arrows: erosion and denudation of cartilage, arrow heads: matrix vertical fissures, circles: superficial fibrillation. Bar = 500 µm (A). OARSI scores of the four groups at indicated time points, which grade histopathology of OA and reflect the lesion depth and extent of OA over the joint. Data were shown as mean ± SD. *p < 0.05 (B).

**Figure 6.**
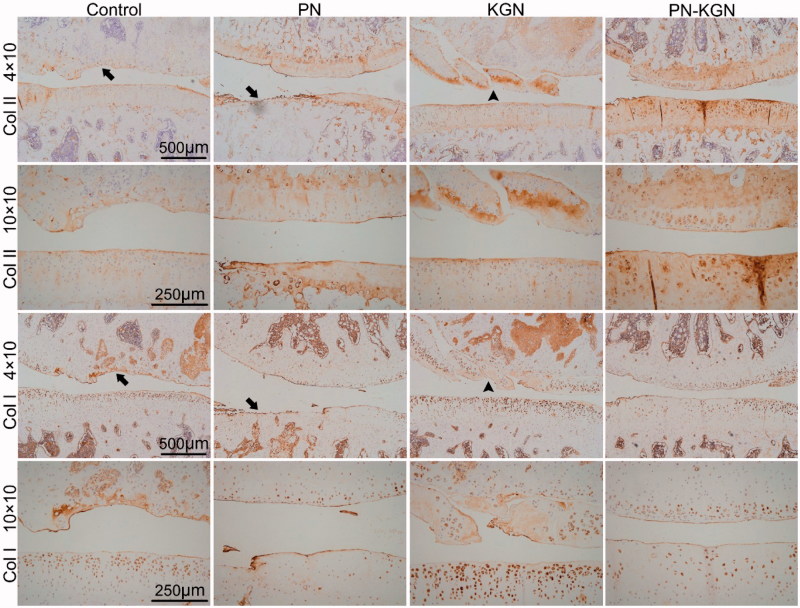
Immunohistochemistry staining for collagen II (Col II) and collagen I (Col I) in Control group, PN group, KGN group and PN-KGN group at 12 weeks. Arrows: cartilage erosion and denudation, arrow heads: matrix vertical fissures. Bar = 500 µm or 250 µm.

The gross appearance of cartilage was evaluated in each group at 3, 6, and 12 weeks. Although there was no obvious macroscopic cartilage abrasion at 3 weeks in each group, cartilage degeneration characterized by fibrillation, surface erosion, pitting, ulceration, and osteophyte formation, as well as subchondral bone exposure, was observed in Control group and PN group at 6 and 12 weeks; however, the rats in PN-KGN group displayed less extensive cartilage destruction at 12 weeks (Figure 4). The macroscopic results were consistent with the histologic staining. Control group and PN group rats showed progressive cartilage degeneration over time with delamination of superficial layers at 6 weeks and surface denudation at 12 weeks. Cartilage in KGN group rats showed minor surface destabilization and thin cartilage at 6 weeks while the cartilage degeneration became obviously at 12 weeks with matrix vertical fissures. By contrast, Cartilage in PN-KGN group rats showed generally intact surfaces and only mild superficial fibrillation even at 12 weeks (Figure 5(A)). Analysis of the OARSI scores revealed no significant differences between Control group and PN group at each time point. However, these two groups had significantly higher OARSI scores compared with KGN group and PN-KGN group at 6 and 12 weeks. Moreover, the OARSI scores were significantly higher in KGN group than that in PN-KGN group at 12 weeks (Figure 5(B)). The metabolism of cartilage matrix was further verified by immunohistochemistry for collagen II (Col II) and collagen I (Col I). Figure 6 showed typical cartilage erosion as well as subchondral bone exposure in Control group and PN group with weak Col II staining. Cartilages of KGN group displayed clusters of chondrocytes and intense Col I staining. However, cartilages of PN-KGN group displayed much stronger Col II-specific staining and less Col I-specific staining compared to other groups at 12 weeks, indicating IA injection of PN-KGN nanospheres could retain the normal compositions of cartilage matrix for a long time.

## Discussion

OA is a common degenerative joint disease which exerts significant detrimental effects on the quality of patients’ life. Commonly prescribed OA medications, such as non-steroidal anti-inflammatory drugs, analgesics and locally administered corticosteroids, only provide symptomatic relief and cannot prevent the development of OA (McAlindon et al., 2014). To attenuate or reverse the progression of OA, researchers have searched for disease-modifying OA drugs to facilitate the regeneration of damaged cartilage (Karsdal et al., 2016). KGN is a characterized small molecule that could free CBF-β from filament A to interact with Runx1, which in turn enhances collagen synthesis (Johnson et al., 2012). KGN has been reported to enhance cartilage regeneration (Li et al., 2016; Shi et al., 2016). It is also reported that KGN help to promote the expression of lubricin, also known as superficial zone protein or proteoglycan 4, which is a multi-level chondro-protective mucinous glycoprotein in joints and confirmed to be essential for repairing joint diseases (Liu et al., 2015).

IA injection is an effective treatment for joints diseases and has several advantages, such as lower total drug doses, initial high local drug concentrations, avoidance of systemic side effects, and fewer drug interactions (Yu & Hunter, 2016). However, small molecules cannot provide adequate therapeutic effects due to rapid clearance and short residence time in joints (Owen et al., 1994). Therefore, development of IA drug delivery systems that can provide sustained release is helpful for successful OA treatment. Several drug delivery systems, such as hydrogel (Singh et al., 2014), liposomes (Stalder & Zumbuehl, 2017), nanoparticles (McMasters et al., 2017), and microparticles (Arunkumar et al., 2016), have been used to achieve sustained release of drugs in joints. In this study, amphiphilic polyurethane with pendant amino group was synthesized to form covalent bonds between the amine group of PN and carboxyl group of KGN, which contribute to sustained and controlled release of KGN *in vitro*.

In terms of formulation design for IA drug delivery, the particle size is an important matter in therapeutic effects because it determines the penetration and retention in cartilage matrix (Kang & Im, 2014). Although there is still controversial about the most appropriate size of drug formulation for IA delivery, Rothenfluh et al. (2008) suggested that self-assembling nanoparticles smaller than 60 nm could be easily transported through the dense collagen network in cartilage extracellular matrix, making cartilage matrix turn from a barrier to a reservoir with nanoparticles penetration. Our designed nanoparticles with a spherical and regular shape and an average size of 25 nm, so that the nanoparticles are expected to efficiently enter the cartilage matrix under the dynamic compression of normal ambulation and therefore could provide an intra-tissue release of KGN rather than just an intra-articular release, offering higher bioavailability in cartilage matrix.

Besides particle size, the charge of particles is another factor affecting the penetration and retention in cartilage matrix (Bajpayee & Grodzinsky, 2017). The negatively charged extracellular matrix inside cartilage provides a unique opportunity to use electrostatic interactions to augment transport, uptake and binding of drug carriers. Positively charged drug-carriers showed faster penetration and higher uptake than their neutral same-sized counterpart (Bajpayee et al., 2014). It is also reported that cationic polymeric nanoparticles had increased retention time and prolonged release profile in joints after IA injection, by forming electrostatic clusters with endogenous hyaluronic acid (Morgen et al., 2013; Kim et al., 2016). As previously reported, pendant amino groups (–NH_2_) could increase the cationic nature of polyurethane (Fang et al., 2014), we speculated that the amino groups could promote electrostatic interactions with anionic proteoglycans and provide longer retention in cartilage matrix.

Biocompatibility is the prerequisite for successful use *in vivo*. The cytotoxicity of PN-KGN nanospheres was first evaluated in chondrocytes and the result indicated that PN-KGN had no cytotoxicity in chondrocytes. A previous study reported that IA injection of nanoparticles gave acute inflammatory responses in mice joints (Vermeij et al., 2015). We evaluated the pro-inflammatory activity of PN-KGN and the test showed that there were no pro-inflammatory activities to chondrocytes *in vitro*. In addition, IA injection of PN did not show joint swelling or aggravate the progress of OA compared to Control group, which reflects the biocompatibility of PN *in vivo*.

Consisted with previous studies (Mohan et al., 2016), IA injection of KGN could attenuate the progress of OA, our study also showed that KGN protected the cartilage at 6 and 12 weeks. However, the cartilage degeneration still became obviously at 12 weeks with matrix loss and vertical fissures. By contrast, IA injection of PN-KGN showed less cartilage degeneration even at 12 weeks. Recently, Kang *et al.* also reported that intra-articular delivery of KGN-conjugated chitosan particles and KGN-contained hyaluronic acid hydrogels could better suppress the development of OA in rats (Kang et al., 2014, 2017). Our results indicate that PN-KGN can more effectively protect articular cartilage and arrest the development of OA than KGN. 

## Conclusion

PN-KGN nanospheres were successfully prepared by synthesis of amphiphilic polyurethane with pendant amino groups and reaction between the amine group of PN and the carboxyl group of KGN to form amide bonds. PN-KGN could continuous release of KGN and shows long-time cartilage protection with less IA injections, which is a better potential strategy to treat OA.
